# Versatile Multi-Functional Block Copolymers Made by Atom Transfer Radical Polymerization and Post-Synthetic Modification: Switching from Volatile Organic Compound Sensors to Polymeric Surfactants for Water Rheology Control via Hydrolysis

**DOI:** 10.3390/nano9030458

**Published:** 2019-03-19

**Authors:** Federico Di Sacco, Andrea Pucci, Patrizio Raffa

**Affiliations:** 1 Zernike Institute for Advance Materials, University of Groningen, AG 9747 Groningen, The Netherlands; f.di.sacco@rug.nl; 2 Dutch Polymer Institute (DPI), P.O. Box 902, 5600 AX Eindhoven, The Netherlands; 3 Department of Chemistry and Industrial Chemistry, University of Pisa, Via Giuseppe Moruzzi 13, 56124 Pisa (PI), Italy; 4 Department of Chemical Engineering, ENTEG institute, University of Groningen, Nijenborgh 4, 9747 AG Groningen, The Netherlands

**Keywords:** atom transfer radical polymerization, multifunctional polymers, polymeric surfactants, VOC sensors, carbon nanotubes nanocomposites, solution rheology

## Abstract

Novel, multipurpose terpolymers based on styrene (PS), tert-butyl methacrylate (tBMA) and glycidyl methacrylate (GMA), have been synthesized via Atom Transfer Radical Polymerization (ATRP). Post-synthetic modification with 1-pyrenemethylamine (AMP) allows non-covalent functionalization of carbon nanotubes, eventually yielding a conductive nanocomposite materials capable of interacting with different Volatile Organic Compounds (VOCs) by electrical resistance variation upon exposure. Moreover, facile hydrolysis of the tBMA group yields polyelectrolytic macrosurfactants with remarkable thickening properties for promising applications in water solution, such as Enhanced Oil Recovery (EOR).

## 1. Introduction

The study and development of functional polymeric materials has attracted growing interest in the scientific and industrial world over the last twenty years, thanks to their wide applicability in many fields of science [1,2]. Their ability to react to different stimuli and conditions from the surrounding environment is surely an appealing feature that drastically improves the versatility for many areas of application, including bio-medical, electronics, catalysis, sensoristics and engineering [3,4,5]. By modifying the monomers composition and the polymerization procedure, it is possible to obtain very different properties and morphologies; in addition, further post-polymerization modification can greatly differentiate the scope for which the polymer was initially meant. In this regard, copolymers play a crucial role. The possibility to link together two or more, monomeric units with different chemical and physical properties and, more interestingly, bearing specific functional moieties, has led researchers to create self-assembly, stimuli-responsive materials [6,7,8,9,10].

The great variety of well-defined morphologies and compositions that could be achieved in polymer science in the last two decades, owe their success to the controlled radical polymerization techniques [11,12], among which Atom Transfer Radical Polymerization (ATRP) is one of the most versatile and robust.

One of the most interesting building blocks in this context is certainly glycidyl methacrylate (GMA). Its epoxide group can be functionalized by ring opening reactions in mild condition using a wide range of nucleophiles like amines, thiols, azides, alcohols, while the acrylic backbone copolymerize quite easily via radical processes with other acrylic monomers [13,14,15,16]. Several polymeric architectures can be obtained by reaction with compounds with different degrees of functionalization, to yield, for example, star and linear polymers [17,18]. Polymeric derivatives of GMA were found able to interact with metal ions, polar and non-polar biomolecules, allowing the construction of drug delivery system with controlled release as function of external stimuli [19,20]. Cross-linked GMA micelles were used as reactors for the synthesis of nanoparticles [21]. Recently, GMA epoxide rings have been functionalized with fluorophores to enable investigation by light emission stimuli-induced structural modifications. Pyrene, Coumarin and Rhodamine B were successfully used to grant to various GMA derivatives emitting properties [16,19,20,21]. Among those, pyrene derivatives has been widely investigated as molecular probes in micellar aggregates or even in the investigation of structural and conformation changes in proteins [22], as a result of its aggregation-induced quenched fluorescence. In addition to the use of pyrene as a probe for structural and conformational changes in-solution, one can take advantage of this moiety to interact with highly conjugated systems, like graphene or carbon nanotubes, thus conferring novel properties to the polymeric system.

**Scheme 1:**
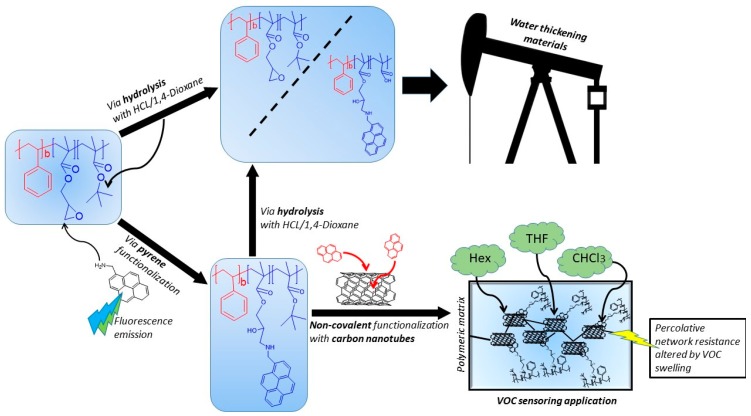
Visual representation of the versatility of the synthesized terpolymers.

The initial aim of this work is to synthesize terpolymers based on diblock poly(styrene-b-tert-butyl methacrylate), coupled with a GMA portion that opens to a large amount of possibility in terms of functionalization and chemical structure. The rationale of such design is that, along with the already discussed possibility to functionalize the epoxy group of GMA, the tert-butyl methacrylate moiety can be easily hydrolysed to methacrylic acid, allowing for applications in water solution and increasing the versatility of the system (Scheme 1).

After synthesis of the terpolymers, pyrene modification of the epoxide ring was performed, to study the ability of the novel functionalized materials to interact and stabilized nanostructured conductive fillers such as multi walled carbon nanotubes and eventually test their use as sensors for volatile organic compound (VOCs). Polymer properties can be modified inherently by changing their chemical composition or by adding functional groups but it is also possible to add fillers, making a composite or nanocomposite material. Carbon nanotubes (CNT) are among the most investigated fillers, as they can enhance the mechanical properties of polymeric materials and, notably, confer electrical and thermal conductivity to the polymer itself. Pyrene moieties have been intensively studied because of their pronounced conjugation that is found to effectively stabilized solutions of CNT in several solvent [23,24,25,26]. Electrical conductivity is of course of great interest among other properties and can be achieved by reaching a critical condition, known as the percolation threshold, where a continuous flow of current passes throughout the whole material, as the concentration of filler reaches a certain needed amount. In these systems, the polymeric matrix can reversibly swell vapour of organic solvents, altering the percolation pattern and thus increasing the resistance through the passage of current. Sensors based on CNT were found to be effective in the detection of the vapour of several organic and inorganic molecules. CNT-based composite materials are extensively used for electrochemical sensing and for VOC detectors and the presence of pyrene can contribute to a good dispersion and stability of the graphitic filler [27,28,29].

Herein, we present a simple, fast and reliable procedure to make polymeric nanocomposite for vapours sensing, using a versatile and easy to synthesize terpolymer that can be furtherly investigated for multiple applications.

To further expand the scope of the materials presented in this work, the pyrene functionalized terpolymers were hydrolysed to achieve novel macrosurfactants-fluorescent-probes that, in principle, could be used as traceable displacing fluids by fluorescent emission monitoring. In addition, the pristine non functionalized terpolymer were hydrolysed as well in order to test their rheological properties for possible applications in water, such as enhanced oil recovery (EOR). The obtained hydrolysed terpolymers structurally resembles diblock copolymers already proposed for EOR applications by our research group [30,31,32,33], with the additional presence of non-charged glycidyl functional groups. This should reduce the polyelectrolyte character of the polymer, providing a better salinity resistance, which is beneficial for the mentioned application. However, this investigation is left at a preliminary stage.

## 2. Materials and Methods

### 2.1. Materials

Styrene monomer (Sigma Aldrich, 99.9%, CAS 100-42-5), N,N,N′,N′′,N′′ pentamethyldiethylenetriamine (PMDETA, Sigma Aldrich, 99%, CAS 3030-47-5), methyl 2-bromopropionate (2-MBP, Sigma Aldrich, 98%, CAS 5445-17-0) were used as received. Tert-butyl methacrylate (tBMA, Sigma Aldrich, 98%, 200 ppm monomethyl ether hydroquinone as inhibitor, CAS 585-07-9) and glycidyl methacrylate (Sigma Aldrich, 97%, 100 ppm monomethyl ether hydroquinone as inhibitor, CAS 106-91-2) were passed through a basic alumina column and stored under nitrogen before use. Hydrochloric acid (HCl, Sigma Aldrich, 37%, CAS 7647-01-0), toluene (Sigma Aldrich, anhydrous, 99.8%, CAS 108-88-3), methanol (MeOH, Sigma Aldrich, anhydrous, 99.8%, CAS 67-56-1), tetrahydrofuran (THF, Sigma Aldrich, anhydrous, 99.9%, CAS 109-99-9), anisole (Sigma Aldrich, anhydrous, 99.7%, CAS 100-66-3), ethanol (EtOH, Sigma Aldrich, reagent grade, CAS 64-17-5), ethyl acetate (EtOAc, Sigma Aldrich, anhydrous, 99.8%, CAS 141-78-6), hexane (HEX, Sigma Aldrich, anhydrous, 95%, CAS 110-54-3) glacial acetic acid (Sigma Aldrich, natural, 99.5%, CAS 64-19-7), diethyl ether (Sigma Aldrich, anhydrous, 99.7%, CAS 60-29-7), 1,4-dioxane (Sigma Aldrich, anhydrous, 99.8%, CAS 123-91-1), dichloromethane (DCM, Sigma Aldrich, anhydrous, 99.8%), chloroform-d (Sigma Aldrich, 99.8 atom % D, CAS 865-49-6), trifluoroacetic acid (TFA, Sigma Aldrich, 99%), sodium carbonate (Sigma Aldrich, 99%), aluminium oxide (Alumina, Sigma Aldrich, neutral and basic, CAS 1344-28-1), ammonium hydroxide (NH_4_OH, Sigma Aldrich, CAS 1336-21-6), multi-walled carbon nanotubes C-150-P (Bayern Material Science, diameter 6–9 nm, length 500 nm, 95%) were used as received. CuBr and CuCl catalysts were stirred in glacial acetic acid at room temperature for 6 h. After a given time, the grey-powdery solid was collected on filter paper using a Buchner apparatus, then washed three times with several mL of acetic acid, ethanol and ethyl acetate and dried under vacuum for 15 h and stored at −16 °C. 1-pyrenemethylamine hydrochloride was converted to the primary amine through a neutralization process with NH_4_(OH) followed by liquid-liquid extraction. Methanol was used as the solvent and toluene as the extractor: a bright yellow solid was recovered after drying in rotary evaporator.

### 2.2. Synthetic Procedure, Functionalization, Hydrolysis and Neutralization of Terpolymers

#### 2.2.1. Synthesis of Polystyrene Macroinitiator (PS-Br)

PS-Br macroinitiator was synthesized according to a reported procedure [30]. The controlled radical polymerization was carried out in bulk or in THF solvent at 100 °C for 3 h. The preparation of PS1 in bulk is reported as an example: 20 mL of styrene and 0.83 g of CuBr were poured under nitrogen in a 100 mL three neck round bottomed flask with a magnetic stirring bar, previously purged with nitrogen. Then 0.64 mL of 2-BMP were added and the apparatus was put in an oil bath set to a temperature of 100 °C. After few minutes, 0.38 mL of PMDETA ligand was added with a syringe to start the polymerization. After the given time, the reaction was stopped by cooling down and addition of 10 mL of fresh THF. Then the mixture was passed through a neutral alumina column to remove the catalyst and eventually precipitated two times in a twenty-fold excess of methanol. The white solid was dried at 70 °C under vacuum for at least 24 h. Characterization was performed with Fourier transform infrared spectroscopy (FT-IR), proton nuclear magnetic resonance (^1^H-NMR) and gel permeation chromatography (GPC); yield was calculated by gravimetric analysis as weight %.

#### 2.2.2. Synthesis of Terpolymer polystyrene-block-(glycidyl methacrylate-co-tert-butyl methacrylate), PS-b-(GMA-r-tBMA)

Chain extension of the PS macroinitiator were performed based on a reported procedure [30,31]. All synthesis was carried out in anisole as the solvent (25–50% v/v of anisole, see result and discussion section). A molar ratio of 300:1 between total amount of monomers and macroinitiator was used for almost all polymerization. The relative ratio between monomers was adjusted from 9:1 to 7:3. In a typical experiment, 0.33 g of PS-Br macroinitiator were put in a three neck round bottomed flash, previously purged with 3 cycles of vacuum/nitrogen, with 9 mg of CuCl catalyst. Then anisole was added under flux of nitrogen and the whole mixture was stirred until complete dissolution of the macroinitiator. Addition of 6 mL of tBMA and 1.7 mL of GMA was performed with a syringe under nitrogen flux. After a few minutes of stirring, the apparatus was put in an oil bath set to 90 °C and finally 0.06 mL of PMDETA initiator was added. After a given time, heating was stopped and 10 mL of THF were added into the flask, then the solution was passed through a neutral alumina column and precipitated in a twentyfold excess of methanol: to the ratios reported in the results and discussion section. milliQ-water 2:1 or n-hexane. The obtained polymers were filtered on paper with a Buchner apparatus, washed several times with hexane and eventually dried at 50 °C under vacuum for at least 24 h. Characterization was performed with IR, ^1^H-NMR and GPC; yield was calculated by gravimetric analysis as weight %. For other experiments, the amounts of reactants and reagents were varied, according.

#### 2.2.3. Kinetic Experiments

The monomer conversion and molecular weights variations during polymerization were assessed via ^1^H-NMR and GPC respectively. A sample of 0.05 mL of the reaction mixture was taken under nitrogen with a syringe at a given time. For the ^1^H-NMR analysis, this amount was diluted in 1.3 mL of CDCl_3_ and cooled down with liquid nitrogen. For the GPC analysis the same procedure was followed using THF instead of CDCl_3_.

#### 2.2.4. Functionalization of PS-b-(GMA-r-tBMA)

The epoxide ring of the GMA portion of the previously synthesized polymers was functionalized by nucleophilic substitution by two amine-derivate compound such phenethylamine (PEA) (model compound) and 1-aminomethylpyrene (1-AMP). Functionalization with PEA. Reactions were tested in THF and dimethylacetamide (DMA) at temperatures of 60 °C and 90 °C. Eventually, DMA at 90 °C was found to be optimal for our purpose. Around 0.5–1 g of terpolymer was dissolved in THF or DMA in a three neck round bottom flask and afterwards the apparatus was purged from oxygen with three cycles of vacuum/nitrogen. PEA (0.09–1 mL) was added in the reaction flask in different molar excess to the amount of GMA functional groups present in the corresponding terpolymer. Molar excesses of 10:1, 5:1, 2.5:1 and 1:1 were tested. After a given time, ranging from 24–72 h, the reaction was stopped by cooling down and the solution was precipitated in a tenfold excess of milliQ-water. Then the solid was filtered with a Buchner apparatus and washed three times with milliQ-water. The recovered solid was dried under vacuum at 60 °C for 48 h. Characterization was performed with EA (Elemental Analysis), FT-IR and ^1^H-NMR.

#### 2.2.5. Functionalization with 1-AMP

Reaction was carried out using 100 mL of DMA as solvent in a round-bottomed flash, under nitrogen atmosphere. A small amount of silica gel has been used as catalyst to improve the ring opening reaction on the GMA portion. Approximately 0.13–1.8 g of terpolymer, 0.02–0.3 g of 1-AMP and silica gel (0 to 15% weight of silica over weight of polymer) were added into the reaction flash followed by three cycles of vacuum/nitrogen to purge from oxygen. Then 5–15 mL of DMA was added under nitrogen flux. Following the complete dissolution of both solid, the flask was heated at 90 °C for the needed time. Finally, the solution was precipitated two times in a tenfold excess of acidic water and filtered on a Buchner funnel. The yielded solid was washed several times with some mL of acidic water and dried 60 °C under vacuum for 48 h. The obtained solid (yellow solid flakes) was strongly emissive under UV irradiation at 366 nm. Characterization of the functionalized polymers (called TP-AMP) was performed with EA, FT-IR and ^1^H-NMR, UV-VIS, Fluorescence spectroscopy, DSC, TGA.

#### 2.2.6. Hydrolysis and Neutralization of PS-b-(GMA-r-tBMA) and TP-AMP

Tert-butyl and epoxy groups of the prepared terpolymers have been hydrolysed to achieve the desired solubility in water. Two different hydrolysis reactions have been carried out as reported in previous works [30,31]; using TFA in DCM solvent and HCl in 1,4-Dioxane solvent. Both acids were added in a large molar excess (10:1) compared to the molar amounts of tert-butyl groups in each polymer. A tenfold excess of both solvents was used as well. About 0.1–0.5 g of polymer was dissolved in 5–50 mL of DCM or 1,4-Dioxane in a 50 mL round bottomed flask under stirring. For the HCl/Dioxane procedure, a reflux condenser was used. Around 0.5–5 mL of TFA or HCl was carefully added to the flask and the reaction was left proceeding at room temperature for 10–16 h for the TFA/DCM reaction and at 100 °C for 3–6 h for the HCl/Dioxane. In the TFA/DCM procedure, the precipitation might happen during a reaction caused by change in solubility [31]; the solid was recovered by filtration in a Buchner apparatus and washed three times with fresh DCM. In the HCl/Dioxane procedure, the solution were precipitated in a twentyfold excess of milliQ-water as the polymer are not readily soluble in neutral water in their electrolytic form [31]. The solid was filtered in a Buchner apparatus and washed several times with fresh milliQ-water. Finally, the polymers were dried under vacuum at 40 °C for about 48 h. The products (called HYD-TP/HCLDIOX or HYD-TP-PEA/HCLDIOX or HYD-TP-AMP/HCLDIOX) were obtained as sticky white solid and were characterized via ^1^H-NMR and FT-IR. The newly formed acid chains were neutralized to increase solubility in water and to enhance the thickening property of the polymers [1,3,33] by addition of an inorganic base followed by dialysis to remove the excess. About 0.1–3.0 g of polymer was added in a 250 mL flask with a magnetic stirring bar and then a large excess of milliQ-water saturated with sodium carbonate was added. To help dissolution, the flask was put in an oil bath set to 75 °C of temperature for about 16–24 h. After the given time, dialysis was performed putting the sealed membrane inside the largest possible excess of dialysing solvent (milliQ-water in our case) with a gentle stirring for 4 days. Dialysing solvent was changed every 8 h. After this procedure, the dialysed polymer was recovered by freeze drying for 72 h. The polymer, yielded as a white soft foam-like solid, was stored at room temperature without any further treatment.

For rheology measurements, the polymers were dissolved in milliQ water to a given concentration. The mixture was left stirring for 10–16 h to achieve full dissolution of the polymer.

### 2.3. Nanocomposite and VOC Exposure Setup Preparation

About 20 mg of AMP-TP were added to 1.5 mL of chloroform into a vial. To facilitate polymer dissolution each sample was ultrasonicated for 10 min at 400 W and 24 kHz with a probe sonicator model UP 400 S by Hielscher Ultrasound. An H3 probe with titanium tip of 3 mm diameter and 100 mm length was used. An ice bath was used to prevent solvent evaporation during sonication. Then, the required amount of MWCNT was poured into the vial and ultrasonicated a second time for 10 min, with the same procedure mentioned above. Eventually, the solution was left to stand for 24 hours. The mixture was then centrifuged with an IEC (model CWS 4236, ThermoFisher Scientific, Hillsboro, OR, USA) machine for 1 h, 4500 rpm at room temperature. The supernatant liquid was then recovered with a Pasteur pipette and eventually transferred into a test tube and sealed.

VOC sensors were fabricated by casting the dispersion onto gold electrodes supported on an integrated device provided by Cad Line (Pisa, Italy). The device is composed of glass fibres woven into an epoxide resin, which grants chemical inertia. In each case, the dispersion was left to evaporate under a fume hood and then dried under vacuum in a Schlenk tube for 4–6 h. This step was repeated between each test to ensure complete solvent removal. The solid dispersions were connected to a digital multimeter (KEITHLEY 2010, Tektronix, Beaverton, OR, USA) and the measured resistances were obtained as a mean from one hundred measurements as allowed by the multimeter settings. The percolation threshold of the composite was assessed using dispersions with different wt.% MWCNTs content. The resistance response of the MWCNT-AMP-TP composite to VOCs was tested with an apparatus built in the laboratory. Sealing of the chambers was allowed by rubber stripes glued to the movable door. Two small holes on backside and top of the chamber permitted the connection wires to pass through the wall (backside) and the solvent to be dropped inside (top). The device bearing the dispersion was stuck to the inside back wall, with the circuit exposed to the interior space. The room volume was set to 4.6 L in all measurements. In the kinetic measurement, the deposition was exposed to a large excess of organic solvent, until complete saturation of the chamber and then resistance values were taken every five minutes for 1 h. A control measurement was performed, prior to every experiment, by measuring the resistance without the presence of solvent. For each deposition three solvent were tested: THF, CHCl_3_ and Hexane. In the first sets of experiments, the desired amount, usually 200 mL, was put in a beaker and placed inside the closed chamber to test the response over time in a saturated environment. To account for the sensitivity of the devices, dispersions were exposed to an increasing amount of solvent. Around 23 μL of solvent (equal to 5 ppm to the chamber volume) was dropped from the top hole using a Gilson pipette and quickly the hole was closed with a rubber septum. Resistance values were taken every minute for a total of twenty and afterwards a new addition of 5 ppm was done until 100 ppm was reached.

### 2.4. Characterization and Instruments

Proton nuclear magnetic resonance (^1^H-NMR) spectra were recorded using a Varian Mercury Plus 400 MHz spectrometer (Varian Inc, Palo Alto, CA, USA).

Fourier transform infrared spectroscopy (FT-IR) spectra were recorded with a Perkin Elmer Spectrum 2000, in Attenuated Total Reflection (ATR) mode.

UV-Vis absorbance spectra were recorded with a Perkin Elmer Lambda 650. This analysis was used to estimate the amount of 1-AMP reacted with the terpolymer. Dilute solutions of free 1-AMP were prepared at different molar concentrations ranging from 10^−3^ to 10^−7^. A calibration curve was built by plotting the absorbance value at 345 nm versus molarity (mol/L) for each solution; eventually a linear behaviour was fitted. The molar amount of 1-AMP reacted was calculated by putting the registered absorbance value (345 nm) into the calibration curve by extrapolating the related molarity value. The conversion level of the GMA epoxide group was evaluated by subtracting the moles of 1-AMP reacted to the initial moles of GMA present in the polymer.

Fluorescence measurements were collected using Fluorolog Horiba Jobin Yvon spectrophotometer (Horiba Jobin Yvon, Kyoto, Japan) equipped with a 450 W xenon arc lamp and single and double-grating excitation and emission monochromators, respectively.

Thermal degradation of the materials and functionalized amount of MWCNT in the AMP-functionalized polymers were analysed via thermogravimetric analysis (TGA) with a TA Q 5000 instrument (TA Instruments, New Castle, DE, USA) under nitrogen flux. All samples were tested in the temperature range of 25 °C to 700 °C with a scan rate of 10 °C/min.

Differential Scanning Calorimetry (DSC) was used to determine the glass transition temperature of some TP and AMP-TP. A TA-Instruments Q1000 DSC system was used for these measurements under a nitrogen flux. Each sample was firstly heated from 20 °C to 150 °C and backwards in order to remove the thermal history of the polymer, at a rate of 10 °C/min.

The viscoelastic behaviour of some hydrolysed polymers in water solution was evaluated via rheology measurements. Dynamic viscosity response to different shear rate was tested at room temperature. Temperature sweep tests were done at constant stress to determine the viscosity response to temperature in the range of 20 °C to 90 °C. Oscillation frequency sweep tests were done at a constant stress to establish the regime of viscoelastic response. Instrument Haake Mars III rotational rheometer was used to perform the test.

Gel permeation chromatography (GPC) measurements were carried out with a HP1100 machine (Agilent Technologies, Waldbronn, Germany) from Hewlett Packard equipped with three 300 mm × 7.5 mm PLgel 3 μm MIXED-E columns in series equipped with a GBC LC 1240 RI (refractive index) detector (GBC Scientific Equipment Pty Ltd, Victoria, Australia). The samples were eluted with THF at a rate of 1 mL/min, at 140 bar of pressure and 40 °C. Molecular weights and PI were determined using the software PSS WinGPC Unity from Polymer Standard Service. Polystyrene standards were used for calibration.

Scanning electron microscope (SEM) analysis was performed using a SEM with environmental mode FEI Quanta 450 ESEM FEG (ThermoFisher scientific, Hillsboro, OR, USA) with an accelerating voltage of 30 kV. The MWCNTs/polymer samples were ultrasonically dispersed in chloroform for analysis. The suspensions were deposited on a gold-coated silicon wafer and allowed to dry in a vacuum system overnight. The wafer was then mounted onto a stainless steel sample holder using carbon tape.

## 3. Results and Discussion

### 3.1. ATRP Synthesis of PS-b-(tBMA-co-GMA) Terpolymer

**Figure 1:**
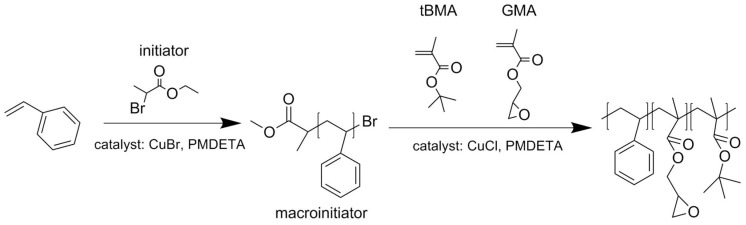
Reaction scheme of the synthesized polymer: ranging from the PS macroinitiator to the final terpolymer.

The polymers synthesized in this work were designed to have a short PS block and a variable block containing tBMA and GMA in a random distribution (assuming similar reactivity of the two acrylic monomers), with different lengths and compositions. The reaction scheme is reported in Figure 1. First, preparation of the polystyrene macroinitiator was carried out using CuBr and PMDETA as the catalytic system and methyl 2-bromopropionate as the initiator. A degree of polymerization of 30 monomeric units was targeted by using of a 30:1 molar ratio between monomer and all other components; this allows to achieve small hydrophobic chains useful for forming micellar-like structure, for the intended application in water. Table 1 indicates the experimental conditions and reagents used for polystyrene ATRP synthesis.

**Figure 2:**
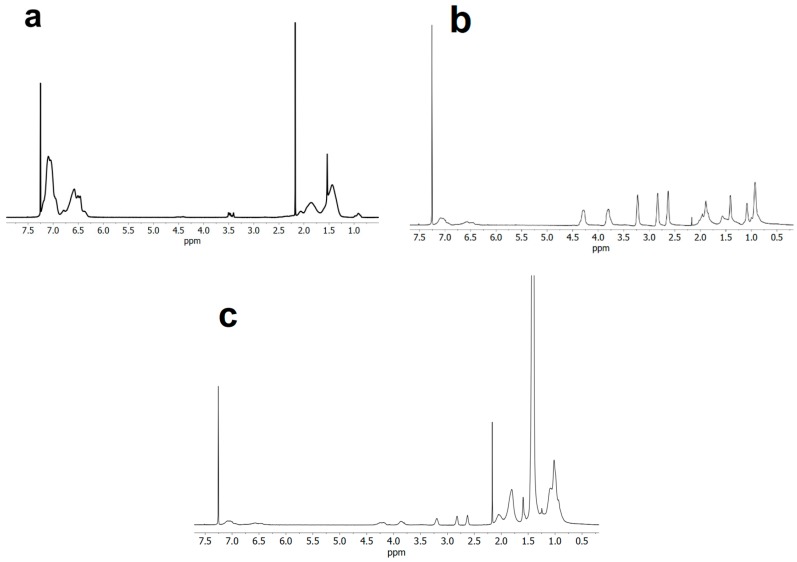
^1^H-NMR (CDCl_3_-d solvent) of macroinitiator PS1 (**a**), terpolymer TP6 (**b**) and terpolymer TP8 (**c**).

Characterization via ^1^H-NMR of macroinitiator PS1 is shown in Figure 2a: characteristic peaks associated with benzene rings occur from 6.25 ppm to 7.20 ppm and two peaks at 1.4 ppm and 2.8 ppm relative to the polymer aliphatic backbone confirm the styrene polymerization. The presence of the ATRP initiator is demonstrated by the 3.4 ppm peak that represents the α-methyl group of its ester moieties. Degree of polymerization was calculated by GPC analysis, showing the typical bell-shaped curve with no presence of shoulders of any sort, suggesting a simultaneous chain growth (See Supporting Info, Figure S1). The fact is also asserted by the low polydispersity index of around 1.1. A number average molecular weight of around 3700 g/mol was obtained for sample PS1 which eventually confirm the presence of 33 repeating unit in the polymer. A lower molecular weight for sample PS2 was obtained as consequence of solvent dilution, needed to control the development of heat reaction as high volume of monomer was used. According to these results, sample PS1 was used as macroinitiator for the ATRP chain extension reactions with mixtures of tert-butyl methacrylate and glycidyl methacrylate. For the chain extension reaction, a molar ratio of 300:1 of total GMA and tBMA monomers related to all other reactants was used for all experiments except for sample TP9 (Table 2), where a 150:1 ratio was tested. Proper reaction conditions needed to be found in order to have good conversion of both monomers and good control over the polymerization. The temperature proved to be a critical parameter. Different temperatures of 30 °C, 60 °C and 90 °C were used while finding the best reaction condition for our purpose. A summary is shown in Table 2. The reaction performed at 90 °C yielded insoluble polymers in most common organic solvents (THF, CHCl_3_, acetone, toluene) probably because of the possible formation of cross-links, either via ring opening of the epoxide groups or chain transfer reactions [34]. On the other hand, reaction performed at 30 °C yield a ready soluble polymer due to the milder conditions, as can be seen by the ^1^H-NMR of Figure 2b with the presence of two typical sharp peaks of epoxide rings proton at 2.63 and 2.84 ppm for the methylene, at 3.22 ppm for the methane group and finally at 3.80 ppm and 4.28 ppm for the methylene protons. However, almost no sign of the diagnostic peak at 1.44 ppm for the tBMA is present, suggesting a too low reactivity at that temperature. Eventually, the reaction at 60 °C was found to deliver a more balanced molar composition to the polymer, as can be seen from Figure 2c which shows a rather intense peak for the tert-butyl group of the tBMA. Degree of polymerization was calculated by use of an ^1^H-NMR procedure reported elsewhere [35]. For all polymers, aromatics peaks of polystyrene were used as reference, because the number of repeating units of the macroinitiator was known in advance from GPC. For GMA calculation, peaks at 2.63 ppm and 2.84 ppm where taken, while the peak at 1.44 ppm was considered for tBMA. Table 2 shows the results of these calculation. However, we should consider here that due to overlapping of NMR signals, presence of impurities and inherent NMR errors, this estimate is not sufficiently accurate, especially for polymers with higher amounts of tBMA. Indeed, for some entries there is a big discrepancy between GPC and NMR data. The GPC data should be considered as the most accurate and will be used for comparisons among polymer samples. Experiment performed at 90 °C of temperature show a predominance of tBMA on the overall molar composition and high polydispersity index higher than those generally observed with ATRP, suggesting a not perfect control. Reducing the temperature to 60 °C was found to be the best option, as a remarkable reduction on the PDI is achieved along all performed synthesis.

### 3.2. Kinetic Analysis

**Figure 3:**
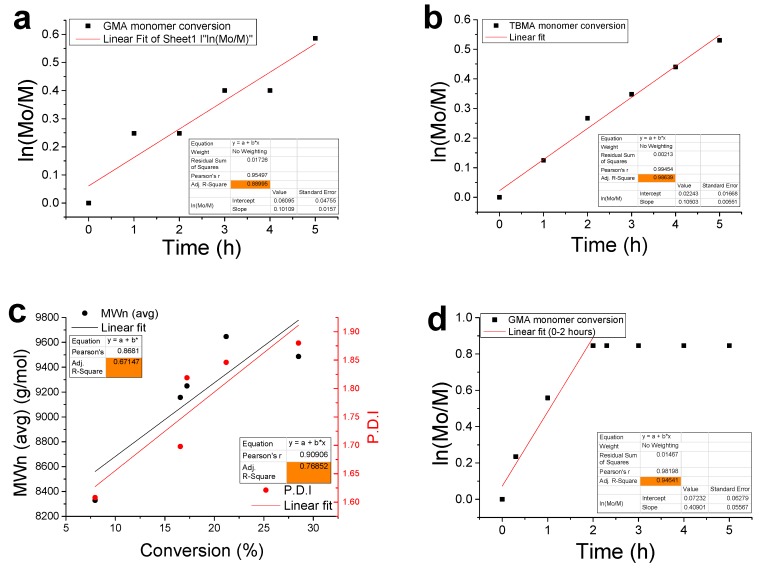

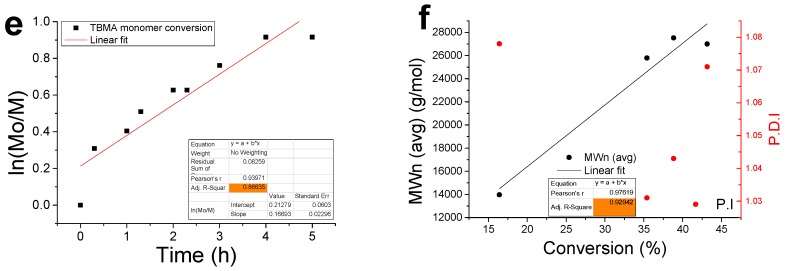
Kinetic plots of: GMA conversion (**a**), tBMA conversion (**b**) and polydispersity index and Mn (**c**) for terpolymer TP9; kinetic plot of GMA conversion (**d**), tBMA conversion (**e**) and polydispersity index and Mn (**f**) for terpolymer TP11.

Kinetic studies were carried out to evaluate the extent of control of the polymerization process in our optimal conditions at 60 °C. Samples were taken from the reaction mixture and conversion of the monomers was calculated by ^1^H-NMR, while molecular weight was monitored by GPC. For calculation via ^1^H-NMR the monomers double bonds peaks (around 5.45 to 6.20 ppm) area decrease was monitored as the reaction proceeded. Areas were normalized compared to the area of anisole methyl group (3.82 ppm). Sample TP9 and TP11 were tested as a different monomer-to-reactant ratios were used, of 300:1 and 150:1, respectively. Figure 3a,b shows, for terpolymer TP9, that the monomer conversion follows in good approximation a first-order kinetics, in accordance with common ATRP synthesis of methacrylic monomers [36]. Using each GPC chromatogram report, PDI and average number molecular weight were plotted as function of conversion, as shown in Figure 3c. the PDI increases with time and the values are somewhat higher than those expected by an ATRP process. This can be due to incomplete initiation of the macroinitiator. Molecular weight shown a fairly linear increase, except for the last data point, at the highest conversion.

A kinetic experiment with a lower monomer to initiator ratio of 150:1 was done for sample TP11; a faster pace in the chains grow can be expected, causing higher conversion and higher PDI. Figure 3d,e show the conversion for both GMA and tBMA monomers respectively. Within the first two hours, both monomers reacted very fast and overall without showing a linear behaviour, except that in the early stages of polymerization. Conversion and PDI values, as expected, are higher compared to the higher 300:1 molar ratio. Figure 3f shows the PDI and average number molecular weight plotted versus monomer conversion: molecular weight increases sharply before two hours of reaction but no linear behaviour is present. In addition, the PDI values obtained for this experiment shows a variation from 1.03 to 1.08, which seem too low for the present case, thus they should not be considered as reliable data. In conclusion, high monomer to initiator ratio of 300:1, along with a reaction temperature of 60 °C, lead to an overall better control on the polymerization. A further investigation on the effect of an even higher molar to initiator ratio was behind the aim of this work.

### 3.3. Functionalization with 1-Pyrenemethylamine (1-AMP)

Several terpolymers were functionalized at 90 °C using DMA as the solvent; these conditions were found to be optimal in experiment performed on a model compound such as phenylethylamine (PEA). To facilitate the ring opening reaction of GMA, different amounts of silica gel were used as catalyst [37]. The results are shown in Table 3. The reaction proceeds without any excess of 1-AMP and in presence of small amount of silica gel as catalyst in 24 and 48 h. Interestingly, the reaction seems to not proceed without use of silica gel, as confirmed by samples TP9-PYR(0) and TP9-PYR(1). The incorporation of pyrene was confirmed by ^1^H-NMR (Supporting info, Figure S6) and visually by shining a UV light on the polymers. In order to quantify the reacted 1-AMP, elemental analysis was carried out, along with an UV-VIS quantitative method (see Supporting Info).

**Figure 4:**
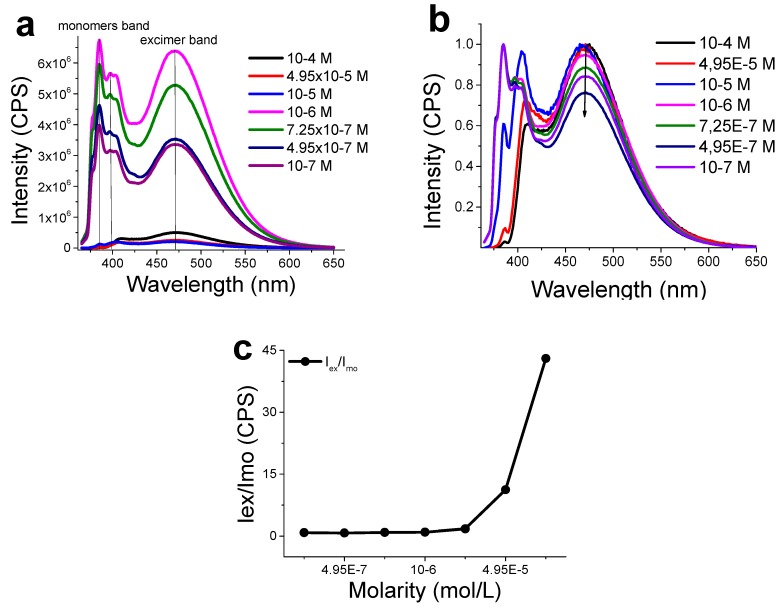
(**a**) Overlay of fluorescent emission spectra at different molarities of TP9-PYR(6) in toluene. (**b**) Normalized emission intensity. (**c**) Ratio between excimer and monomer emission as function of molarity.

Spectroscopic characterization of the novel AMP-functionalized polymer in solution was carried out by fluorescence spectroscopy, using dilute solution of the polymer in toluene. Polymer TP9-PYR(6) was selected for most of our experiments, being the sample with the highest amount of functionalized 1-AMP. In Figure 4a peaks assigned to pyrene monomer emission are present at 375 nm and 396 nm, related with the S_2_→S_0_ and S_1_→S_0_ pyrene transition respectively and also a significant red-shifted band at 480 nm is present at all concentrations with the highest intensity recorded at 10^−6^ M and attributed to the formation of excimers [38,39]. More concentrated solutions (10^−4^ to 10^−5^ M) show quenched emission possibly due to the aggregation-caused quenching behaviour of the pyrene chromophore [40,41]. In Figure 4b, normalized spectra by their maximum value display how different bands change in intensity with molarity. Notably, the progressive increase of molar concentration causes the prevalence of the excimer band with respect to that of pyrene monomers as also is clearly visible in Figure 4c, where the ratio between excimer and monomer emission (taken respectively at 470 nm and 348 nm) is plotted versus the molarity [42].

Absolute quantum yields as high as 6.6% were also calculated, thus confirming the aggregation-caused quenching behaviour of the pyrene labelled polymer samples gathered from emission spectra.

### 3.4. CNTs Dispersion and Stabilization by AMP-Functionalized Terpolymer

**Figure 5:**
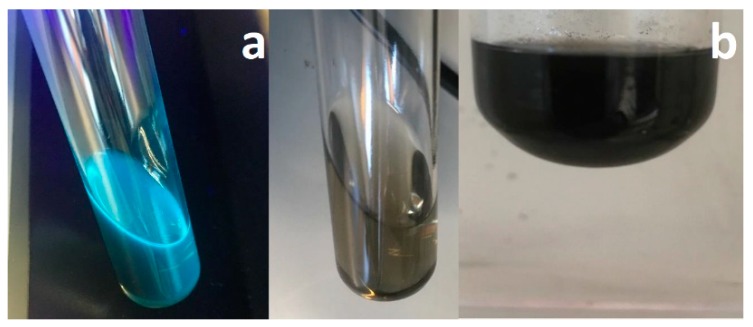
(**a**) Chloroform dispersion of TP9-PYR(6) and CNTs after centrifugation and recovery. On the left: sample under UV light (366 nm). On the right: the same dispersion without UV illumination. (**b**) Dispersion of TP9-PYR(6) and CNTs after 1 month in chloroform.

**Figure 6:**
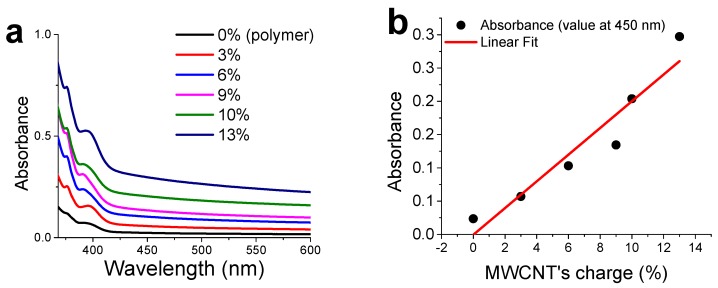
(**a**) UV-Vis spectra of dispersion at different weight concentrations of CNTs. (**b**) Absorbance values (taken at 540 nm) versus CNTs charge, the red line is the applied linear fit.

Dispersions of CNTs in AMP-functionalized polymer TP9-PYR(6) (26% mol AMP) in chloroform were made at different weight concentrations of CNT and a fixed amount of polymer (20 mg/1.5 mL). As shown in Figure 5, the dispersion remains stable for at least one month, suggesting an effective interaction between polymer and nanotubes. UV-Vis analysis was carried out as the mono dimensional graphitic layer produces a strong light scattering, proportional to the amount of CNTs dispersed [43]. Several dispersions with different weight concentrations of CNT ranging from 3% to 13%, as well with a solution with the pristine polymer, were tested. A nearly linear behaviour is observed by plotting the absorbance versus the CNTs charge at a fixed wavelength of 450 nm (Figure 6). TGA was also used to estimate the effective amount of non-covalently functionalized CNTs by comparing the residue at 700 °C of each dispersion with the one of the pristine polymer. The residual amount correlates well with the CNT concentration. The amount of CNT estimated by TGA are reported in Table 4.

### 3.5. Scanning Electron Microscopy (SEM) Analysis of CNTs Dispersion

**Figure 7:**
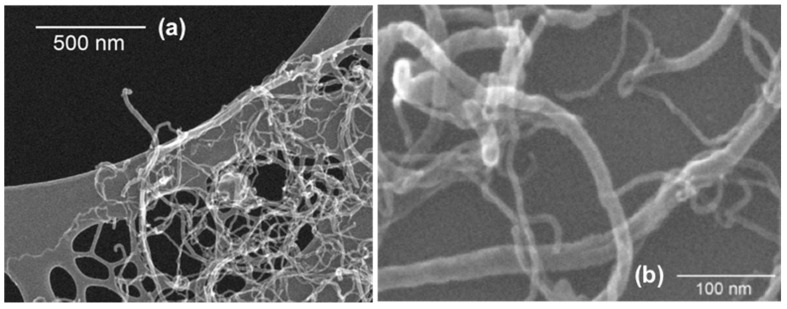
SEM images of 10% CNTs dispersion with TP9PYR(6) at different magnifications. (**a**) scale bar 500 nm; (**b**) scale bar 100 nm.

To investigate the quality of CNTs’ exfoliation, the dispersion with the highest loading of CNT (D10, Table 4) was analysed by SEM. Micrographs in Figure 7 show secondary electron images of the dispersion confirming an effective exfoliation of the nanotubes provided by the interaction with the polymer matrix and with no sign of detectable aggregation. Moreover, the average length of multi-walled carbon nanotubes MWCNTs is comparable to their nominal length (1–10 mm), thus suggesting that the CNTs are not severely damaged by the exfoliation process.

### 3.6. Percolation Threshold Calculation

**Figure 8:**
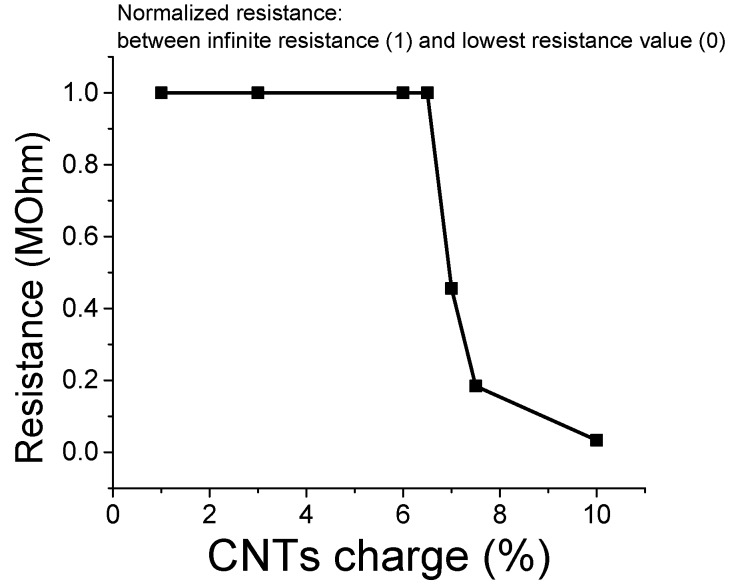
Percolation threshold for MWCNTs dispersion in AMP-functionalized polymer PYR6. In the Y axis an arbitrary range of resistance (in MOhm).

The electrical behaviour of the composite was evaluated by depositing the dispersion via solution casting over an electrical circuit. The device was then connected to a digital multimeter with a data logger. Only resistance was measured, as the thickness affected by a substantial error. The graph regarding the percolation threshold (Figure 8) was made as follows: the sample with infinite resistance (no conduction) was assigned to a value of 1 MOhm and placed on top of the Y axis. The percolation threshold was determined at a concentration in weight of about 6–7%. Notwithstanding this value appearing higher than those of the state of the art dispersions in acrylic functionalized polymers [44,45], the designed system proposed in this work appears to provide a faster and cheaper procedure for promptly investigating the resistive features of the electrically-conductive composites. Samples with concentrations of 7.5% and 10% exhibit resistances of 184 kOhm and 33 kOhm, respectively, and suggest the formation of an effective percolation pathway within the polymer matrix.

### 3.7. Volatile Organic Compound (VOCs) Exposure Experiments

**Figure 9:**
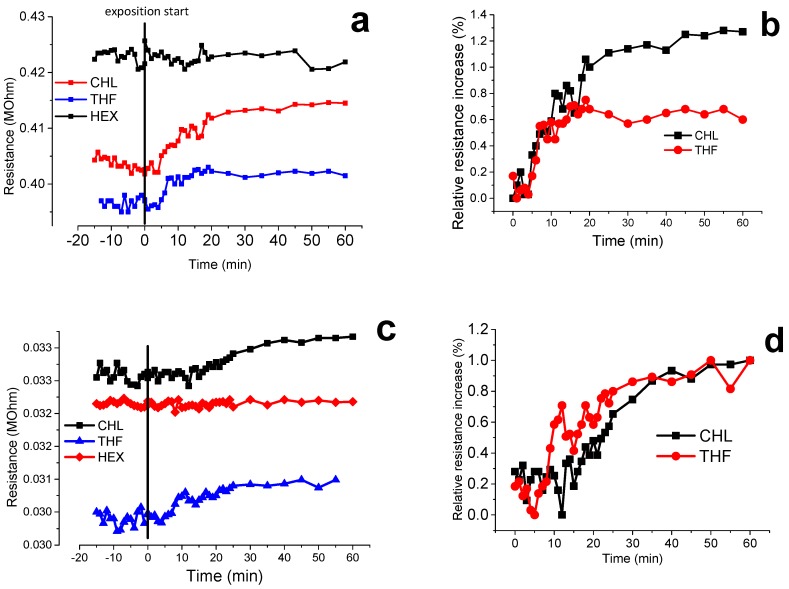
(**a**) Resistance of 7 wt.% MWCNTs/ TP9PYR(6) dispersion with the exposure time towards saturated atmospheres of chloroform, tetrahydrofuran and hexane for 1 h and (**b**) relative resistance variation (%) towards saturated atmosphere of chloroform and tetrahydrofuran; (**c**) the same of a) but testing 10 wt.% MWCNTs/ TP9PYR(6) dispersion; (**d**) the same of b) but testing 10 wt.% MWCNTs/TP9PYR(6) dispersion.

Electrically conductive MWCNTs/ TP9PYR(6) polymer dispersions were used as a small sensing device for three different VOCs with diverse affinities with acrylates-based materials. As soon as VOC molecules reached the sensing surface, polymer matrix swelling occurs thus yielding a potential variation of the CNTs percolation pathways and a variation of the composite resistance with the exposure time. As can be seen in Figure 9a, exposure to CHCl_3_ and THF for a device containing 7 wt.% of CNTs produce a sensible increase of resistance. The effect is more pronounced in the first 10–20 min after the exposure. Then, after an initial quick increase, saturation is reached and the resistance levelled off reaching a plateau [46]. As expected, HEX exposure did not provide any sensible variation. A comparison between CHCl_3_ and THF is reported in Figure 9b, with the former showing a resistance increasing of 2.5%, compared to the 1.7% by using the latter as VOC. This was expected as CHCl_3_ is a better swelling solvent for the acrylic polymeric matrix [47]. The same experiments were carried out on the device with 10% wt.% of CNTs (Figure 9c–d). In this case, the higher concentration of CNTs inside the polymer forms a more stable percolation pathway that is less perturbed by solvent exposure.

**Figure 10:**
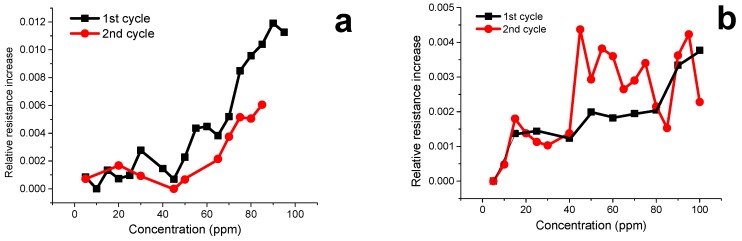
Relative resistance variation (%) of 7 wt.% MWCNTs/TP9PYR(6) dispersion as a function of (**a**) CHCl_3_ and (**b**) THF vapours concentration (ppm).

The sensibility of the sensor containing 7 wt.% of CNTs was then investigated by using progressive amount of solvents up to 100 ppm. Notably, for both THF and CHCl_3_ the device starts to become sensitive for VOC concentration higher than 30–40 ppm. After the first exposure to VOCs, the vapours were desorbed and the system was then analysed to determine possible variation of the resistance response. A comparison between two successive exposure cycles of CHCl_3_ is displayed in Figure 10a, with the first cycle giving an overall variation in resistance of about 3%, while the second display a slightly lower variation of 1.6%, both determined at the maximum vapours concentration (100 ppm). Same experiments were performed using THF as the solvent, as shown in Figure 10b, resulting in a similar trends in terms of reduced resistance variation for the second cycle, thus suggesting a loss of sensitivity after the first VOC exposure according to similar nanocomposite devices already investigated in the literature [48]. Nevertheless, the system is able to discriminate different VOCs, according to the solvent affinity with the polymer matrix.

### 3.8. Hydrolysis and Neutralization of TP and AMP-Functionalized Polymers

To investigate the versatility of the prepared polymers for applications in water solution, hydrolysis of the tert-butyl group was performed according to two procedures described in the experimental section. The procedure with HCl in 1,4-Dioxane was preferred due to an easier recovery of the polymer after precipitation. The completeness of the reaction was confirmed by ^1^H-NMR in d6-DMSO (Supporting info, Figure S3), that shows disappearance of the t-butyl group and formation of a broad peak ascribed to OH groups formed by opening of the epoxide ring. FT-IR also clearly shows peaks for the OH groups (Supporting Info, Figure S4). ^1^H-NMR also shows that pyrene groups are still attached to the polymer (Supporting Info, Figure S5).

**Figure 11:**
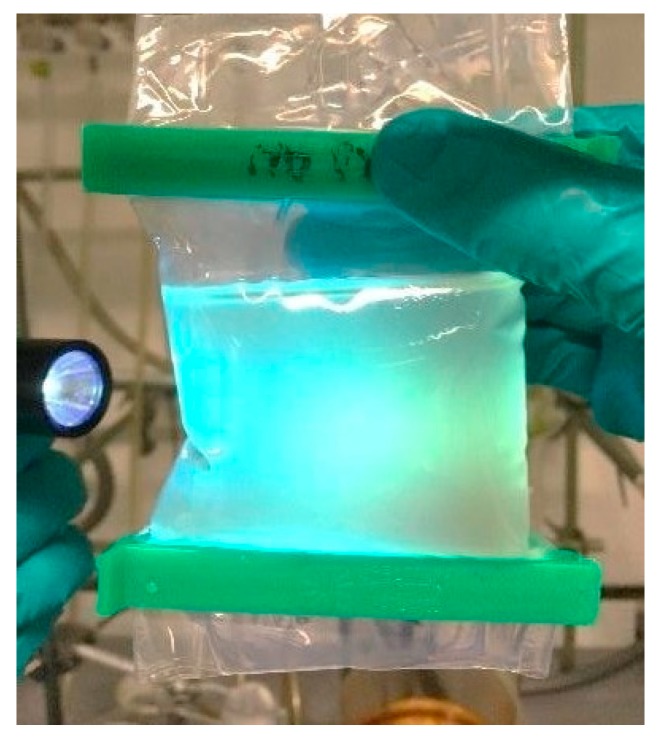
AMP-functionalized polymer dissolved in a dialysis membrane under the excitation at 366 nm.

Acidic chains of the hydrolysed polymers were neutralized using a base as described in the experimental section, then solutions were prepared to test the rheological properties. The pyrene-functionalized polymers, however, despite showing good solubility (see Figure 11) did not show the desired thickening ability upon dissolution in water.

Most likely, the presence of an easy aggregating moiety like pyrene can induce an intramolecular folding of the extended, soluble, chains. Formation of π-π stacking interaction between different pyrene molecules or with the internal styrene units could hinder the gelation process by causing a collapsed micellar structure. Even at a relatively high concentration of polymer (up to 3 wt.%) the polymer solution did not present a significantly high viscosity. This explanation is partially confirmed by the fluorescence emission data registered previously, showing a high level of pyrene aggregation at low concentrations in a good solvent like toluene. It is then reasonable suggesting that aggregation phenomena can even more easily happen in a hydrophilic environment. However, deeper investigations are needed to confirm these suggestions. However, hydrolysed terpolymer without pyrene groups effectively showed the desired thickening behaviour, along with a quick solubility in water.

### 3.9. Rheological Measurements

**Figure 12:**
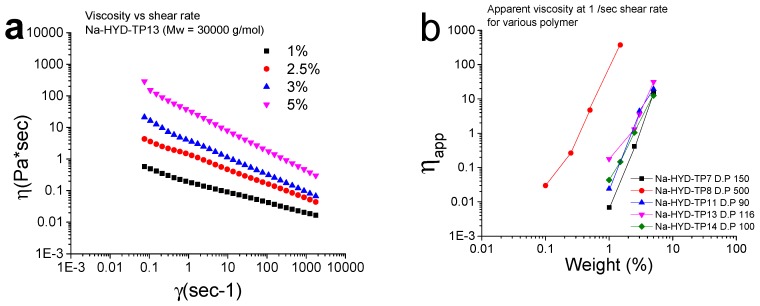

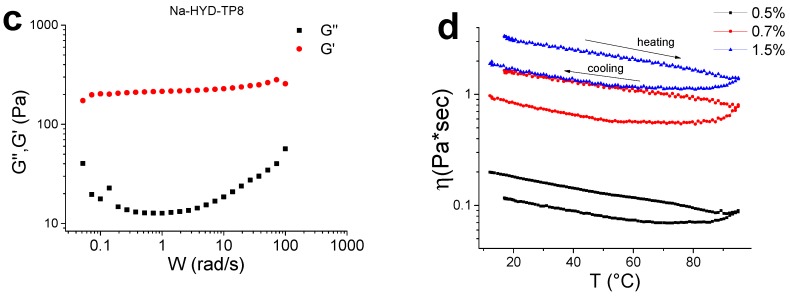
Shear viscosity measurements for various polymer: (**a**) Na-HYD-TP13 and (**b**) apparent viscosity taken at 1 sec^−1^ for all analysed polymers; (**c**) oscillation frequency sweep for Na-HYD-TP8 and (**d**) viscosity dependence of temperature in range 20 °C to 90 °C for polymer NaHYD-TP8 at different weight concentration.

Rheological measurements were carried out on several neutralized-hydrolysed (Na-HYD) polymers. Shear viscosity at different weight concentrations, ranging from 1% to 5%, were measured, the results are reported in the Supporting Info, Figures S6–S9. As shown in Figure 12a, the investigated polymer present good thickening properties, with viscosity much higher than water at all concentration and along the whole investigated range of shear. All solutions show shear thinning behaviour and no Newtonian plateau in the observed range. This is in line with what observed for analogous systems [30,31,32]. It was not possible to investigate the behaviour at lower frequencies, to see if Newtonian regime would ever occur, due to limitations of the rheometer in the low-frequencies range. The shear thinning is more pronounced at higher concentrations. In the investigated range, the molecular weight seems to have little to no effect on the viscosity of the measured samples. Analogous block copolymers not containing the GMA moieties, exhibit high viscosity already at 1 wt.% concentration [30,31,32] As expected, the presence of neutral monomers in the hydrophilic block causes a comparably lower degree of stretching in solution, lowering the viscosity. On the other hand, these systems should be less sensitive to salinity (not tested in this work).

Figure 12b displays the apparent viscosity versus concentration reported at a frequency of 1 sec^−1^. Comparing samples at different molecular weights and similar compositions (TP11 vs TP13 and TP14), it can be observed that the hydrophilic chain’s length does not seem to have a significant influence. Contrarily, the ratio between acrylic acid and glycidyl groups has a major effect: polymer NaHYD-TP8, having the highest relative amount of acrylic acid units (almost 10:1), shows a significantly higher viscosity at the same concentration, compared to all the other studied polymers.

Viscosity values are slightly higher than water (10^−3^ Pa*sec at 20 °C) even at very low concentration eventually suggesting a possible use in EOR application. Frequency sweep measurements were made to test the double, elastic and viscous, response of the materials. Tested samples (Figure 12c) have G’ > G’’ in the whole range of investigation, showing the formation of a strong viscoelastic gel at concentration of 0.1 wt.%. In addition, the elastic modulus is almost independent from the frequency, indicating a pronounced solid-like material [49,50]. As observed for viscosity, at lower frequencies the limit of the instrument is reached and the data points become more scattered, so it is not possible to observe a crossover frequency, which in this case will be very low, indicating a long relaxation time. Further results on frequency sweep are reported in the Supporting Info, Figures S10–S12. As shown in Figure 12d, viscosity response to temperature produces a steep and constant reduction on the viscosity. Notably, concentration does not affect the behaviour of the materials as all curves show the same variation with temperature.

## 4. Conclusions

In this work, the synthesis of multipurpose terpolymers, based on styrene, tert-butyl methacrylate and glycidyl methacrylate, is reported. Block copolymers, constituted by a hard hydrophobic block of polystyrene and a soft, hydrolysable, acrylate block, were prepared via ATRP, which allowed the obtaining of polymers with different compositions and molecular weights and a narrow molecular weight distribution. Kinetic studies showed good control over the polymerization of the two acrylate monomers on the PS macroinitiator.

The presence of epoxy groups from the glycidyl methacrylate allowed an easy post-synthetic modification with 1-pyrenemethylamine. These polymers showed a good non-covalent interaction with MWCNTs and the derived solid dispersions provided electrically-conductive nanocomposite materials, with the characteristic of a selective electrical sensor for volatile organic compounds. Moreover, facile hydrolysis of the tert-butyl and glycidyl groups yielded polyelectrolytic macrosurfactants with remarkable thickening properties for promising application in Enhanced Oil Recovery (EOR). The relative amount of free carboxylic groups and glycidyl groups affected the thickening ability of the investigated polymers, apparently to a larger extent than the molecular weight. The pyrene-functionalized polymers did not lose the fluorescent group upon hydrolysis, making them suitable as fluorescent probes in water, for example to track polymer adsorption during oil recovery processes. However, they did not display good thickening ability in water solution after hydrolysis, probably due to chain collapse in solution, caused by the strong hydrophobic interactions of the pyrene groups. Overall, the presented results evidenced the versatility of the prepared polymers, both for dry and wet smart applications.

## Figures and Tables

**Table 1:** Conditions used for ATRP synthesis of two poly(styrene) macroinitiator.

Sample	[Sty]:[I]:[C]:[L]	Sty (mL)	Solvent (mL)	Mn ^1^ (g/mol)	Time (h)	Yield (%)	Sty Unit	PDI
PS1	30:1:1:1	20	Bulk	3700	1.5	55	33	1.1
PS2	30:1:1:1	40	20 (toluene)	2500	3	45	21	1.1

^1^: Determined by GPC.

**Table 2:** Summary of ATRP chain extension reactions performed using PS1 as macroinitiator and average numeral molecular weight calculation.

Polymer	Molar Ratio ^1^	Solvent (Anisole) Volume %	Time (h)	T (°C)	Mn (GPC) g/mol	Mn (NMR) g/mol	PDI ^2^	Sty-GMA-TBMA Units
TP1	1:1:1:270:30	25	18	90	26,400	23,000	1.2	33-27-239
TP2	1:1:1:270:30	25	4	90	18,700	41,300	1.6	33-23-155
TP3	1:1:1:210:90	25	2	90	11,800	45,500	1.85	33-99-197
TP4	1:1:1:210:90	25	1	90	14,700	17,200	1.59	33-52-45
TP5	1:1:1:210:90	25	0.5	90	11,300	19,650	1.47	33-21-39
TP6	1:1:1:270:30	50	48	30	12,900	20,200	1.36	33-109-9
TP7	1:1:1:270:30	50	5	60	35,100	29,700	1.09	33-33-152
TP8	1:1:1:270:30	50	15	60	31,900	118,000	1.13	33-57-531
TP9	1:1:1:210:90	50	5	60	29,600	12,400	1.25	33-37-26
TP10	1:1:1:255:45	50	5	60	24,400	27,150	1.1	33-23-144
TP11	1:1:1:105:45	50	10	60	15,400	18,700	1.67	33-31-85
TP12	1:1:1:210:90	50	8	60	30,700	48,900	1.15	33-133-196
TP13	1:1:1:210:90	50	8	60	32,500	27,450	1.12	33-63-116
TP14	1:1:1:210:90	50	5	60	30,900	24,500	1.14	33-49-106

^1^: [I]:[CuCl]:[PMDETA]:[tBMA]:[GMA]; ^2^: Polydispersity Index.

**Table 3:** AMP functionalization reaction carried out in DMA solvent.

Sample	GMA Molar Amount in the Polymer	AMP: Polymer (Molar Ratio)	SiO_2_ w/w on Polymer	Reaction Time (h)
TP9-PYR(0)	36%	1.0	0%	48 ^1^
TP9-PYR (1)	36%	2.5	0%	48 ^1^
TP7-PYR (2)	16%	1.5	10%	48
TP9-PYR(3)	39%	1.5	5%	24
TP11-PYR(4)	24%	1.0	10%	24
TP11-PYR(5)	24%	1.0	7.5%	48
TP9-PYR(6)	39%	1.0	7.5%	48
TP13-PYR(7)	31%	1.0	7.5%	48
TP13-PYR(8)	31%	1.0	7.5%	48
TP14-PYR(9)	28%	1.0	5%	48

^1^: no reaction.

**Table 4:** CNTs effective charge registered from the two-dispersion sequences.

Sample	CNTs Feed (%)	Average Residue (%)	CNTs Average Effective Charge (%)
TP9-PYR(6)	0%	7.3	0
D3	3%	9.8	2.4
D6	6%	13.0	5.6
D75	7.5%	13.9	6.5
D8	8%	14.9	7.5
D9	9%	16.0	8.5
D10	10%	15.9	8.5

## Supplementary Materials

The following are available online at https://www.mdpi.com/2079-4991/9/3/458/s1, Figure S1: GPC of macroinitiator PS1; Figure S2: UV-Vis spectra, registered in toluene solvent, used for the calibration curve method; Figure S3: Calibration curve made from free pyrene dilute solution; Figures S3 and S4: ^1^H-NMR and FT-IR for hydrolysed terpolymer HYD-TP11; Figure S5: ^1^H-NMR spectrum of a hydrolysed AMP-based terpolymer; Figures S6–S9: Shear viscosity measurements for various polymers; Figures S10–S12. Loss and Storage moduli in oscillatory frequency sweep of polymers solutions; Table S1: Conversion of GMA moiety from both UV-Vis method and EA methods.
