# Supplementary material for: Versatile Multi-Functional Block Copolymers Made by Atom Transfer Radical Polymerization and Post-Synthetic Modification: Switching from Volatile Organic Compound Sensors to Polymeric Surfactants for Water Rheology Control via Hydrolysis
Source: Nanomaterials (Basel). 2019 Mar 19;9(3):458. doi: 10.3390/nano9030458 (PMC6474081; doi:10.3390/nano9030458)
Supplement: Supplementary file 1 [file nanomaterials-09-00458-s001.pdf]

Supporting Info of the manuscript entitled "Versatile multi-functional block copolymers made by ATRP and post-synthetic modification: switching from VOC sensors to polymeric surfactants for water rheology control via hydrolysis" by Federico Di Sacco, Andrea Pucci* and Patrizio Raffa*

3.1 ATRP synthesis of PS-b-(tBMA-co-GMA) terpolymer

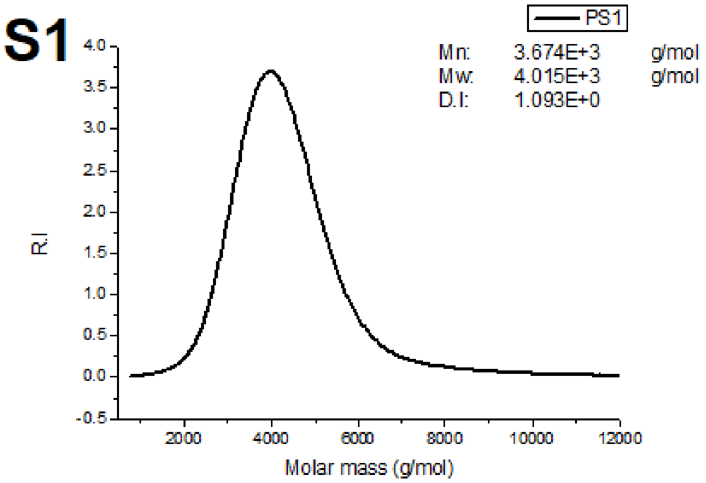

Figure S1. GPC of macroinitiator PS1

3.3 Functionalization with 1-pyrenemethylamine (1-AMP)

UV-Vis quantitative method procedure

Figure S2 display the spectra taken on dilute solution of free 1-AMP and on the functionalized polymers used for this investigation. Characteristic pyrene absorbance peaks at 328 nm and 345 nm are visible and a calibration curve by plot of the absorbance values at 345 nm versus the molarity was gathered and eventually shown in Figure S3. A comparison of the data obtained from EA and UV-VIS calibration are shown in Table S1 which show a good reproducibility of both method, eventually reflecting the possibility of using a fast and simple procedure to calculate the amount of functionalized pyrene. Is important to remember that this method is trustworthy only if the extinction coefficient of the absorbing molecule do not change after functionalization. This assumption is reasonable, as the pyrene moiety is not directly connected to the polymer backbone, but via a spacer.

S2

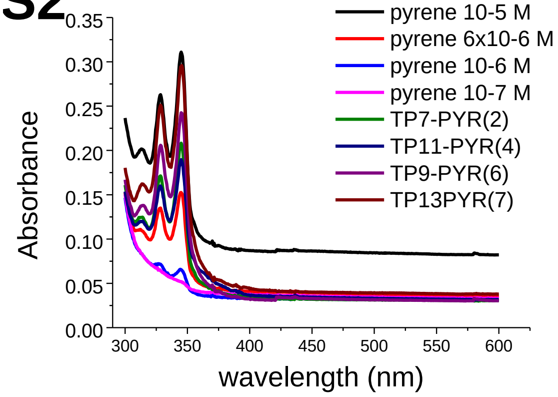

Figure S2. UV-Vis spectra, registered in toluene solvent, used for the calibration curve method. Dilute solution ranging from 10^{-7} up to 10^{-5} were used

S3

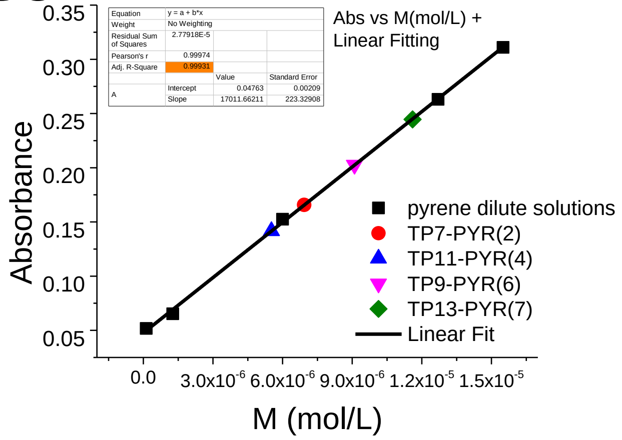

Figure S3. Calibration curve made from free pyrene dilute solution, coloured symbols represent the analysed polymer. Linear fit is shown as a black line.

Table S1. Conversion of GMA moiety from both UV-Vis method and EA methods

Sample	Weighted mg	Expected GMA mol	Expected AMP mol	Conv. Abs vs Molarity	Conv. EA
TP7-PYR(2)	3,5	3,30E-06	1,39E-06	42,1	39,4
TP11-PYR(4)	3,5	7,00E-06	2,76E-06	39,5	38,6
TP9-PYR(6)	3,7	6,65E-06	4,55E-06	68,4	67,7
TP13-PYR(7)	3,7	7,18E-06	5,78E-06	80,5	78,0

3.8 Hydrolysis and neutralization of TP and AMP-functionalized polymers

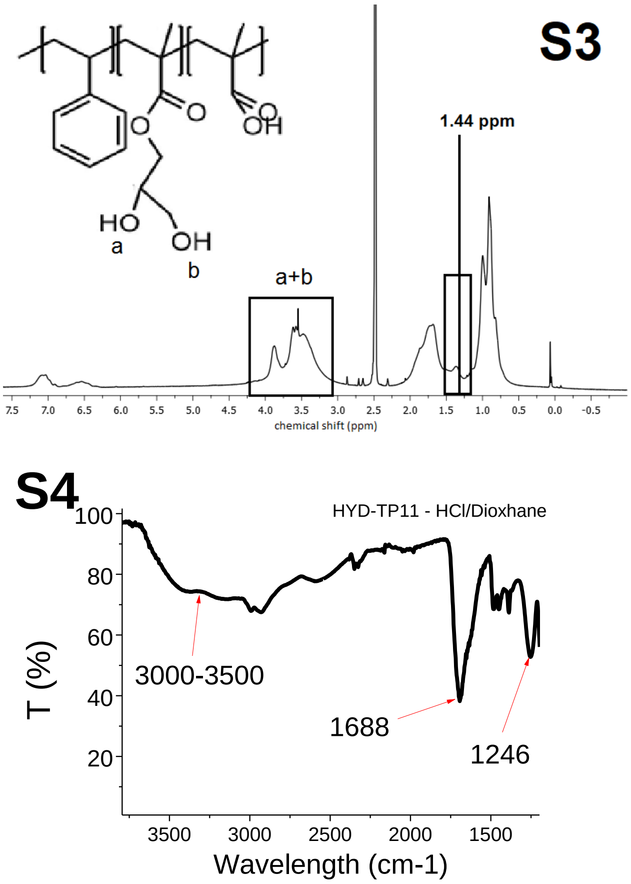

Figure S3 and S4. ^{1}H-NMR (S3) and FT-IR (S4) for hydrolysed terpolymer HYD-TP11.

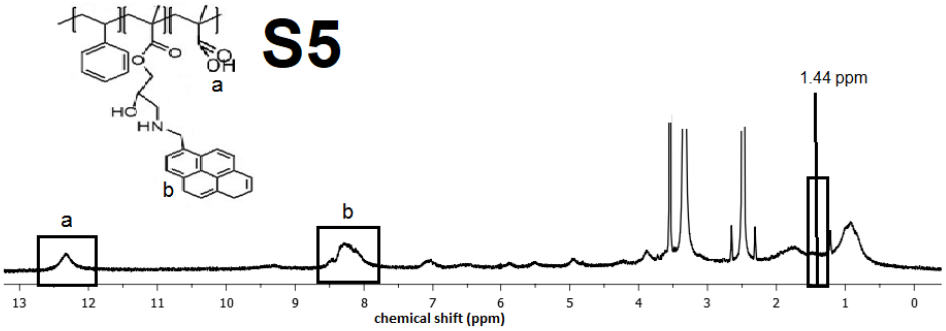

Figure S5. ^{1}H-NMR spectrum of a hydrolysed AMP-based terpolymer

3.9 Rheological measurements

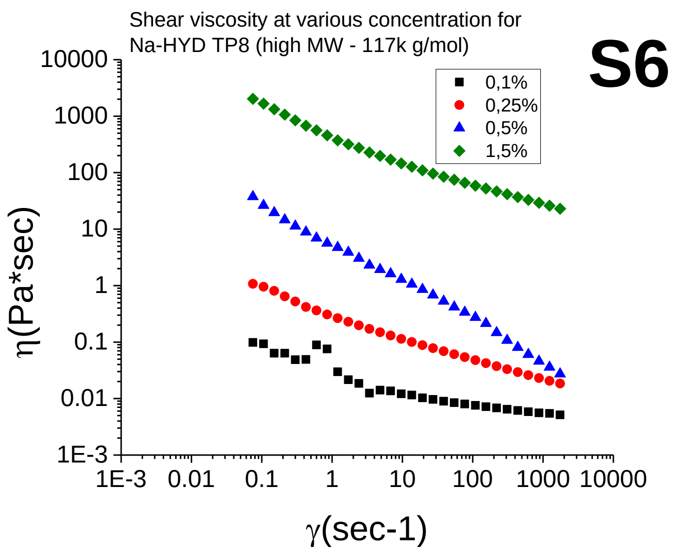

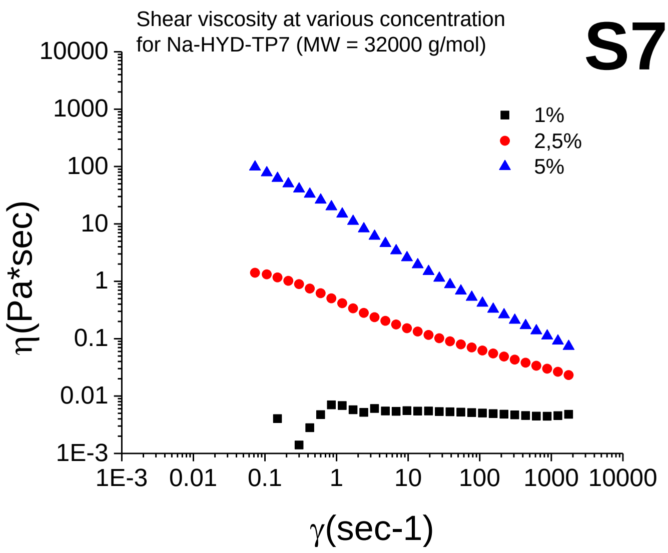

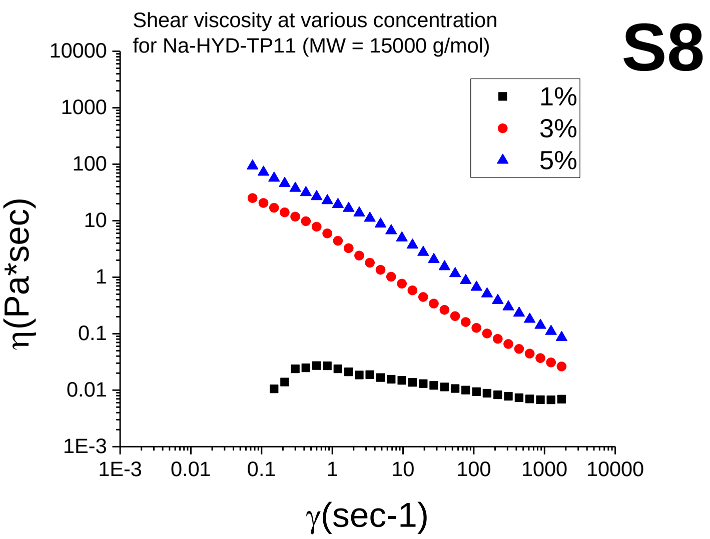

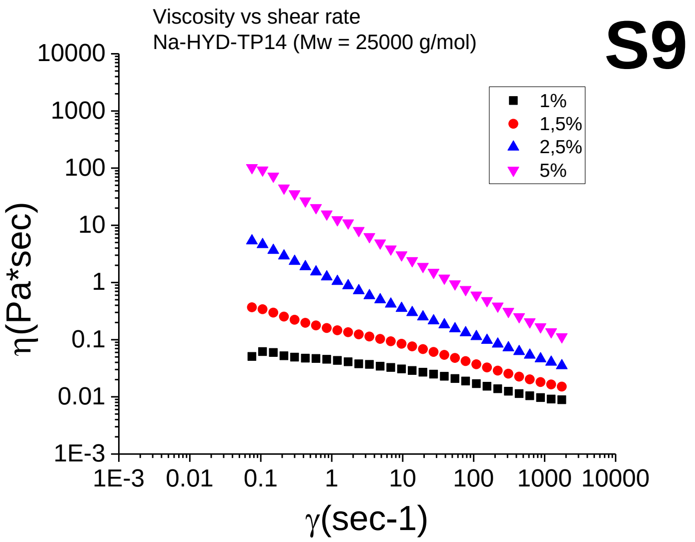

Figure S6–9. Shear viscosity measurements for various polymers: Na-HYD-TP8 (S6), Na-HYD-TP7 (S7), Na- HYD-TP11 (S8), Na-HYD-TP14 (S9)

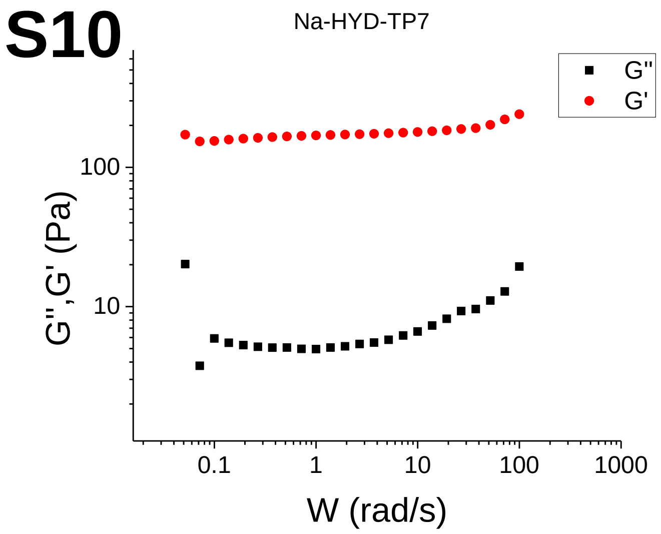

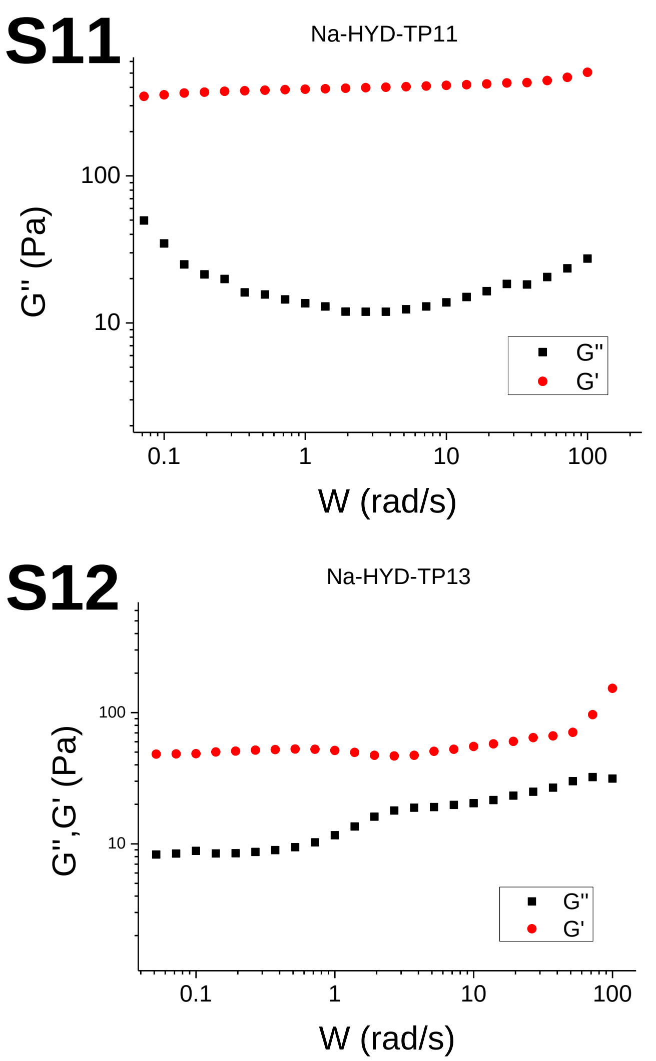

Figure S10–12. Loss and Storage moduli in oscillatory frequency sweep of polymers solutions: Na-HYD-TP7 (S10), Na-HYD-TP11 (S11), Na-HYD-TP13 (S12).
